# Scalable, effective, and rapid decontamination of SARS-CoV-2 contaminated N95 respirators using germicidal ultraviolet C (UVC) irradiation device

**DOI:** 10.1038/s41598-021-99431-5

**Published:** 2021-10-07

**Authors:** Raveen Rathnasinghe, Robert F. Karlicek, Michael Schotsaert, Mattheos Koffas, Brigitte L. Arduini, Sonia Jangra, Bowen Wang, Jason L. Davis, Mohammed Alnaggar, Anthony Costa, Richard Vincent, Adolfo García-Sastre, Deepak Vashishth, Priti Balchandani

**Affiliations:** 1 Department of Microbiology, Icahn School of Medicine at Mount Sinai, New York, NY 10029 USA; 2 Graduate School of Biomedical Sciences, Icahn School of Medicine at Mount Sinai, New York, NY 10029 USA; 3 Department of Medicine, Division of Infectious Diseases, Icahn School of Medicine at Mount Sinai, New York, NY 10029 USA; 4 The Tisch Cancer Institute, Icahn School of Medicine at Mount Sinai, New York, NY 10029 USA; 5 Global Health and Emerging Pathogens Institute, Icahn School of Medicine at Mount Sinai, New York, NY 10029 USA; 6 Smart Lighting Engineering Research Center, Rensselaer Polytechnic Institute, Troy, NY 12180 USA; 7 Department of Electrical Computer and Systems Engineering, Rensselaer Polytechnic Institute, Troy, NY 12180 USA; 8 Department of Chemical and Biological Engineering, Rensselaer Polytechnic Institute, Troy, NY 12180 USA; 9 Center for Biotechnology and Interdisciplinary Studies, Rensselaer Polytechnic Institute, Troy, NY 12180 USA; 10 Department of Biological Sciences, Rensselaer Polytechnic Institute, Troy, NY 12180 USA; 11 Department of Civil and Environmental Engineering, Rensselaer Polytechnic Institute, Troy, NY 12180 USA; 12 Department of Neurosurgery, Icahn School of Medicine at Mount Sinai, New York, NY 10029 USA; 13 Department of Medicine, Section of General Internal Medicine, Icahn School of Medicine at Mount Sinai, New York, NY 10029 USA; 14 Department of Biomedical Engineering, Rensselaer Polytechnic Institute, Troy, NY 12180 USA; 15 BioMedical Engineering and Imaging Institute, Icahn School of Medicine at Mount Sinai, New York, NY 10029 USA

**Keywords:** Infectious diseases, Respiratory tract diseases, Microbiology techniques, Disease prevention, Public health, Microbiology, Diseases, Antimicrobials

## Abstract

Particulate respirators such as N95s are an essential component of personal protective equipment (PPE) for front-line workers. This study describes a rapid and effective UVC irradiation system that would facilitate the safe re-use of N95 respirators and provides supporting information for deploying UVC for decontamination of SARS-CoV-2 during the COVID-19 pandemic. To assess the inactivation potential of the proposed UVC germicidal device as a function of time by using 3 M 8211-N95 particulate respirators inoculated with SARS-CoV-2. A germicidal UVC device to deliver tailored UVC dose was developed and test coupons (2.5 cm^2^) of the 3 M-N95 respirator were inoculated with 10^6^ plaque-forming units (PFU) of SARS-CoV-2 and were UV irradiated. Different exposure times were tested (0–164 s) by fixing the distance between the lamp and the test coupon to 15.2 cm while providing an exposure of at least 5.43 mWcm^−2^. Primary measure of outcome was titration of infectious virus recovered from virus-inoculated respirator test coupons after UVC exposure. Other measures included the method validation of the irradiation protocol, using lentiviruses (biosafety level-2 agent) and establishment of the germicidal UVC exposure protocol. An average of 4.38 × 10^3^ PFU ml^−1^ (SD 772.68) was recovered from untreated test coupons while 4.44 × 10^2^ PFU ml^−1^ (SD 203.67), 4.00 × 10^2^ PFU ml^−1^ (SD 115.47), 1.56 × 10^2^ PFU ml^−1^ (SD 76.98) and 4.44 × 10^1^ PFU ml^−1^ (SD 76.98) was recovered in exposures 2, 6, 18 and 54 s per side respectively. The germicidal device output and positioning was monitored and a minimum output of 5.43 mW cm^−2^ was maintained. Infectious SARS-CoV-2 was not detected by plaque assays (minimal level of detection is 67 PFU ml^−1^) on N95 respirator test coupons when irradiated for 120 s per side or longer suggesting 3.5 log reduction in 240 s of irradiation, 1.3 J cm^−2^. A scalable germicidal UVC device to deliver tailored UVC dose for rapid decontamination of SARS-CoV-2 was developed. UVC germicidal irradiation of N95 test coupons inoculated with SARS-CoV-2 for 120 s per side resulted in 3.5 log reduction of virus. These data support the reuse of N95 particle-filtrate apparatus upon irradiation with UVC and supports use of UVC-based decontamination of SARS-CoV-2 during the COVID-19 pandemic.

## Introduction

Coronavirus disease (COVID-19) is caused by the severe-acute respiratory syndrome coronavirus 2 (SARS-CoV-2). This respiratory pathogen belonging to the beta-coronavirus group emerged at the end of 2019 in the city of Wuhan, Hubei province, China^1^. As of late March 2021, SARS-CoV-2 infected ~ 124 million individuals while accounting for more than 2.5 million deaths worldwide^2^. So far, the United States of America is one of the hardest hit countries worldwide. The long-term public health and economic consequences are alarming and are often compared to the “Spanish-flu” pandemic caused by an influenza virus in 1918 that resulted in more deaths than victims in World War I^3^.

During pandemic outbreaks, frontline workers and essential workers across a spectrum of industries including but not limited to medical professionals, scientists, academics, as well as individuals within food and beverage industries, are required to carry out pivotal duties to keep society functioning. Critical measures employed to prevent the spread of the disease include avoiding social interactions through social distancing and lockdowns in combination with improved hygiene measures. Additionally, the use of personal protective equipment (PPE) such as masks, gloves, sleeves and face-shields play an essential role in protecting front line workers as well as minimizing the spread of the respiratory pathogen SARS-CoV-2^4^. SARS-CoV-2 can spread in aerosol droplets that are formed when an infected person coughs, sneezes or speaks. Advanced filtering facepiece respirators (FFR), such as N95, KN95 and N99 are used as a measure of personal protection when enhanced exposure risk to SARS-CoV-2 is expected, including within patient care settings or diagnostic and research laboratories. As the extent of this pandemic resulted in the long-anticipated shortage of FFR and other facemasks, the need for more PPE supplies across the world escalated significantly^5^. Such a situation negatively impacted healthcare settings, putting frontline workers in a vulnerable position, potentially resulting in a collapse of the healthcare system. This was further corroborated by the World Health Organization (WHO) stating that insufficient supply of such PPEs would put essential frontline workers at the risk of being exposed to SARS-CoV-2, which would further facilitate the spread of this contagious disease^6^. As a measure to address this issue, methods to conserve PPE were discussed by panelists from the Journal of American Medical Association (JAMA) including the suggested reuse of PPE^7^.

Several factors must be taken into consideration when evaluating the suggested reuse of respirators. First, it is important to have a general idea of the persistence of SARS-CoV-2 on surfaces. Several studies have demonstrated that viruses such as SARS-CoV and its phylogenetic-relatives such as Middle-East Respiratory Syndrome virus (MERS) and seasonal coronaviruses (HCoV) may persist on inanimate object surfaces including wood, plastic, metal, paper and glass up to 9 days^8,9^. However, the stability of the virus on surfaces is greatly dependent on other factors such as temperature, humidity and the presence of other proteins in viral droplets^10–14^. In contrast, a recent study suggested that SARS-CoV-2 is only able to last on such surfaces more or less up to 72 h and in some cases up to 28 days based on external factors mentioned above^14,15^. Given the variability of SARS-CoV-2 viral survivorship on different surfaces and the close, long-term contact of reused respirators with the face, a scalable, reliable, cost-effective disinfection procedure that would enable the safe reuse of PPE is of global interest^11^.

Several methods of decontamination have been proposed to facilitate the reuse of PPEs. These typically branch off from chemical methods (i.e., alcohol, bleach, etc.) or energetic methods such as heat inactivation, microwave-based systems or ultraviolet (UV) based systems^11^. In summary, chemical based inactivation methods have indicated that virus contaminated surfaces can be effectively decontaminated using 70% ethanol, bleach solutions (0.1% Sodium Hypochlorite) and hydrogen peroxide (0.5% H_2_O_2_) with enough exposure time^12^. The issues with these methods include inability to scale up to disinfect a large number of units and the need for specialized competencies to work with chemical supplies. Furthermore, several chemical treatment methods have been shown to impair the functionality of the filters found in respirators^16^. In contrast, energy-based systems have also been explored for decontaminating PPEs. One category of methods suggested was heat inactivation in which various protocols such as heating to 70 °C for 24 h or heating for 30 min at 56 °C depending on the model implemented^17,18^. This method is limited in applicability to a subset of respirators belonging to the FFP2 category since most manufacturers recommend against heat treatment due to deleterious effects on the respirator materials which could lead to ineffective protective capacity^16,19^.

Another energy-based decontamination method is the use of germicidal UVC (200–280 nm) based systems^20^. UVC based disinfection methods are effective since viral genetic materials such as RNA (relevant in this case given that SARS-CoV-2 genome is an RNA based one) and DNA strongly absorbs UVC radiation^21,22^. This results in microbial genetic damage at a molecular and structural level (via photodimerization), severely compromising the functionality of the virus leading to viral inactivation, consequently prohibiting the capacity for the virus to replicate upon exposure to compatible hosts^20,23^. Germicidal properties of UVC on PPE and FFRs such as N95s have been studied in the context of other respiratory viral pathogens such as influenza A viruses^24,25^. In particular, the use of UVC lamps to disinfect FFR has been studied by the National Institute for Occupational Safety and Health (NIOSH) and found that FFR retained good filtering and air flow properties using UVC exposures that would far exceed those used to disinfect FFR. This indicates UVC irradiation methods have the potential to be highly effective, point-of-use disinfection systems^26^. UVC radiation is a line-of-sight disinfection system and pathogens in the shadows or shielded from the UVC radiation can remain viable unless reflective UVC is sufficient for inactivation.

Here we evaluate the effectiveness of a rapid UVC germicidal disinfection system that can be scaled up to effectively inactivate SARS-CoV-2 in potentially contaminated respirators. Using test coupons of the 3 M-N95 respirator, we aimed to measure the total exposure time to UVC radiation that would result in adequate log reduction of SARS-CoV-2 to indicate the lack of infective viral particles. We further aimed to demonstrate the inactivation potential of the device by using a model non-enveloped virus. This information provides support for use of UVC-based decontamination of SARS-CoV-2 virus during the COVID-19 pandemic.

## Materials and methods

### UVC respirator exposure system

**Figure 1:**
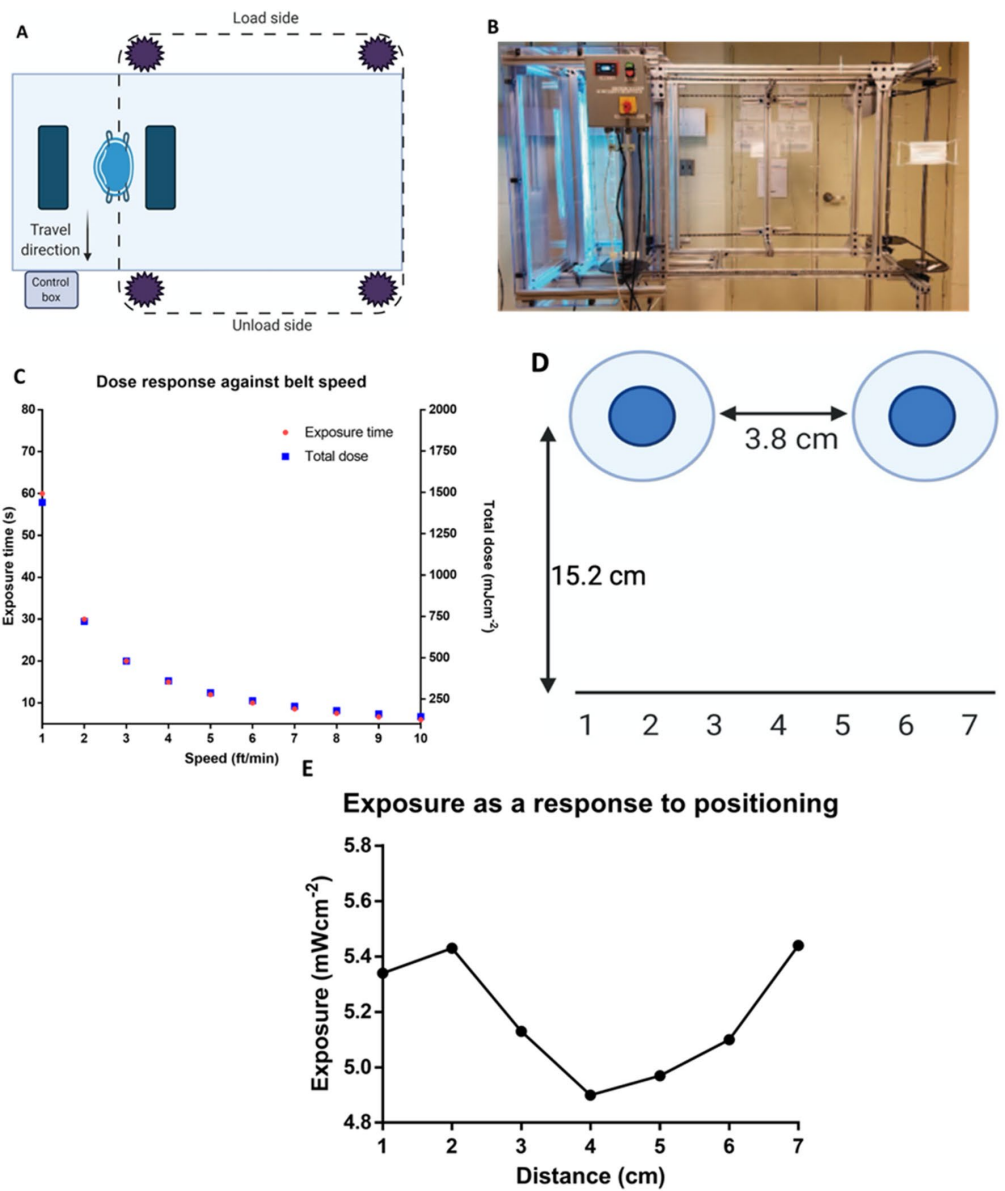
Schematic, dose response and exposure characterization of the proposed UVC decontamination device. (**A**) Schematic of the operational germicidal device. The UVC germicidal device comprises a conveyor belt which is scalable. (**B**) Real image of the prototype germicidal device. (**C**) Changes to the dose response curve as a response belt speed. Total dose changes in response to the conveyor belt speed and exposure time. (**D**) Schematic of the experimental setup lamp and surface positioning. The germicidal lamp bulbs contain 3.8 cm between them and the distance from the lamp to the surface is 15.2 cm. Exposure changes as a result of positioning was monitored. (**E**) Exposure profile of the lamp based on the surface placement as indicated in (**D**). The UVC 254 nm detector was placed in positions 1 cm apart, covering a total distance of 7 cm. Measurements were taken at 3 min at each time point after initial lamp warm up.

A simple UVC respirator exposure system was fabricated from two opposing IP55 high power mercury (Hg) lamp fixtures (American Ultraviolet, Lebanon, Indiana) configured vertically and a simple motorized conveyor system. The prototype system design moves FFR, supported vertically by the straps, between the two lamps where the dose is controlled by the belt speed of the respirator conveyor system. The concept is shown schematically below (Fig. 1A) and a prototype was built, and its mechanical operation tested (Fig. 1B).

The system shown here can expose contaminated N95 respirators at UVC dose levels up to 1.5 J cm^−2^ in an exposure time of approximately one minute (Fig. 1C). The total dose can be controlled by changing the speed of the conveyor system (Fig. 1C). As configured the unit contains two opposing IP55 Hg UVC lamp fixtures, but two more pairs can be added to achieve effective irradiation at higher belt speeds for increasing FFR treatment throughput.

For dose testing with SARS-CoV-2, one lamp was removed from the system (shown in Fig. 1B) and evaluated for germicidal activity in a biosafety-level 3 (BSL-3) facility at the Icahn School of Medicine at Mount Sinai (ISMMS). UVC irradiance was measured using a calibrated Optometer P 9710 Gigahertz-Optik UVC meter (Türkenfeld, Germany) with the 254 nm detection head that was positioned at a fixed distance approximating the distance of the FFR from one of UVC lamps as configured in the UVC mask exposure system. The lamps were allowed to warm up for approximately 2 min to achieve stable UVC output. Due to potential masking effects that may occur in the space between the two bulbs, the changes due to positioning was assessed using 1 cm increments and experiments were done in position number 2 (Fig. 1D,E). To approximate the exposure of a contaminated FFR, tests with the single lamp system were made by exposing experimental respirator-based test coupons, first on one side, then the other, as described below.

### Cells and viruses

Vero-E6 cells (ATCC CRL-1586™, clone E6) were cultured in Dulbecco's Modified Eagle Medium (DMEM; 1X with glucose and sodium pyruvate, Corning 10013CV) supplemented with 10% Fetal Bovine Serum (FBS) from PEAK (PS-FB03), penicillin–streptomycin (100X Corning 30-0002-CL), MEM non-essential amino-acids (100X Gibco; 25025CL) and Hepes buffer (1 M, Gibco; 15630-080) at 37 °C with 5% CO_2_. Cells were passaged every three days until required for experimentation. A549 human lung carcinoma cells (ATCC CCL-185) were cultured in DMEM supplemented with 10% heat-inactivated fetal bovine serum (FBS; HyClone SH0066.03) and penicillin/streptomycin (100 × Corning 30-0002-CL) at 37 °C with 5% CO_2_. Infectious virus assays using 3rd generation lentivirus expressing constitutive enhanced green fluorescent protein (LV-EGFP; Addgene plasmid # 36083) were performed in the biosafety level 2 (BSL-2) Stem Cell Core Facility at Rensselaer Polytechnic Institute (SCCFRPI). Viral stocks were produced using human embryonic kidney (HEK293) cells, with collection and concentration of lentivirus 48 h after transfection of 3rd generation plasmid constructs. Infectious virus assays for this study using SARS-CoV-2 (USA-WA1/202, bei resource—NR52281) were handled within a BSL-3 containment facility at ISMMS by trained personnel upon approval of protocols by the institutional biosafety committee. Propagation of viral stocks were done in Vero-E6 cell confluent monolayers by using an infection medium composed of DMEM supplemented with 2% FBS, Non-essential amino acids (NEAA), Hepes and penicillin–streptomycin at 37 °C with 5% CO_2_ for 72 h. A cell culture adapted murine Encephalomyocarditis virus (EMCV; ATCC VR-12B, non-enveloped virus) was expanded and titrated in Vero-CCL81 cells with DMEM and 2% HI-FBS and penicillin–streptomycin at 37 °C with 5% CO_2_ for 48 h^27^. Viral genomes were sequenced for verification via Oxford nanopore platform.

### Inactivation of N95 respirator test coupons spiked with lentivirus

Test coupons of the 3 M-N95 respirator containing all layers of the 3 M 8211-N95 particulate respirator were cut under sterile conditions resulting in final dimensions of 25 mm × 25 mm. N95 respirator test coupons were loaded with LV-EGFP by incubation in a 50 ml conical tube containing 30 ml Dulbecco’s Phosphate Buffered Saline (DPBS) with 2 × 10^8^ transduction units (TU) of LV-EGFP. Virus was allowed to adsorb into the test coupons for 15 min. Coupons were placed in 10 cm petri dishes (2 dishes, each with 4 coupons). One dish with four coupons was placed, open, under the UVC Hg lamp with aluminum foil under the petri dish to reflect UVC radiation. The second dish was covered, wrapped in aluminum foil, and kept in the same biosafety cabinet without UVC exposure as a control for presence of infectious virus. The UVC Hg lamp was placed 15.2 cm (6 inches) above the coupon surface. Coupons were exposed to UVC radiation for 1 min (equivalent of 2 mJ cm^−2^), turned over with sterile forceps, and exposed to UVC radiation for 1 additional minute. After UVC treatment, coupons were transferred to 15 ml conical tubes and submerged in 5 ml of infection medium (DMEM + 1.5% HI-FBS). Control and UVC-treated infection media were used immediately for viral infectivity assays.

### Infectivity assays

Efficiency of LV-EGFP inactivation was tested by serial tenfold dilutions of infection medium in the 15 ml conical tubes, followed by inoculation of 100% confluent A549 cell monolayers in duplicate. Cells were incubated for 48 h, fixed with 4% paraformaldehyde, treated with 4′,6-diamidino-2-phenylindole (DAPI) nuclear stain for 10 min and imaged with a ThermoScientific Cellomics ArrayScan XTI high-content microscope to visualize infected cells as indicated by green fluorescent protein (GFP). Total cell number was determined by counting DAPI + objects and percent-infected determined by DAPI + nuclei associated with EGFP signal.

### Inactivation of N95 respirator test coupons spiked with SARS-CoV-2 and EMCV

Test coupons of the 3 M-N95 respirator containing all layers of the 3 M 8211-N95 particulate respirator were cut under sterile conditions resulting in final dimensions of 25 mm × 25 mm. These were then loaded with SARS-CoV-2 by incubating them in 50 ml conical bottom tubes containing 30 ml of DMEM spiked with 1 × 10^6^ plaque forming units (PFU) SARS-CoV-2. The virus was allowed to adsorb into the test coupons for 15 min at room temperature. Afterwards, the coupons were transferred to sterile tissue culture grade 10 cm petri-dishes. These were placed over aluminum foil and under the Hg UVC lamp after lamp warm-up. In parallel, a similar setup was conducted in which the spiked dishes were incubated in a biosafety cabinet without exposing to UVC radiation to confirm the presence of virus in the N95 test coupons. A working distance of 15.2 cm was maintained between the UVC Hg lamp and the top of the coupon surface within the petri dish. The UVC irradiance was measured at the working distance to be 5.43 mW cm^−2^ and was measured using the Optometer P 9710 Gigahertz Optik (Türenfeld, Germany) equipped with a cosine corrected 254 nm detector and the coupons were exposed for either 2, 6, 18, 54 120, 164 and 420 s per side and were flipped using sterile forceps. The coupons were then transferred to 15 ml conical bottom tubes and were submerged in 3 ml of infection medium and was vortexed for one minute and were left overnight at 4 °C and were assayed the following day. Inactivation assays for the non-enveloped EMCV virus was conducted using the above protocol using exact conditions in a BSL-2 setting.

### Plaque assays

Confluent monolayers of Vero-E6 cells in 12-well plate format were infected with tenfold serially diluted samples in 1X phosphate-buffered saline (PBS pH 7.0; Corning 21040CV) supplemented with bovine serum albumin (BSA; MP-Bio 810063) and penicillin–streptomycin for an hour while gently shaking the plates every 15 min. Afterwards, the inoculum was removed, and the cells were incubated with an overlay composed of MEM with 2% FBS and 0.05% purified agar (OXOID-UK; LP0028) for 72 h at 37 °C with 5% CO_2_. The plates were subsequently fixed using 4% formaldehyde overnight and the formaldehyde was removed along with the overlay. Fixed monolayers were blocked with 5% milk in Tris-buffered saline with 0.1% tween-20 (TBS-T) for an hour. Afterwards, plates were immunostained using a monoclonal antibody cocktail against SARS-CoV-2 Spike (Creative-Biolabs; 2BCE5) and SARS-CoV-2 nucleocapsid protein NP (Creative-Biolabs; NP1C7C7) at a dilution of 1:1000 followed by 1:5000 anti-mouse IgG monoclonal antibody and was developed using KPL TrueBlue peroxidase substrate for 10 min (Seracare; 5510-0030). After washing the plates with distilled water, the number of plaques were counted. Data shown here is derived from three independent experimental setups. EMCV samples were processed in VERO-CCL81 cells in 6 well format, incubated for 48 h, and developed with crystal-violet.

## Results

### Inactivation of N95 respirator test coupons spiked with LV-EGFP

**Figure 2:**
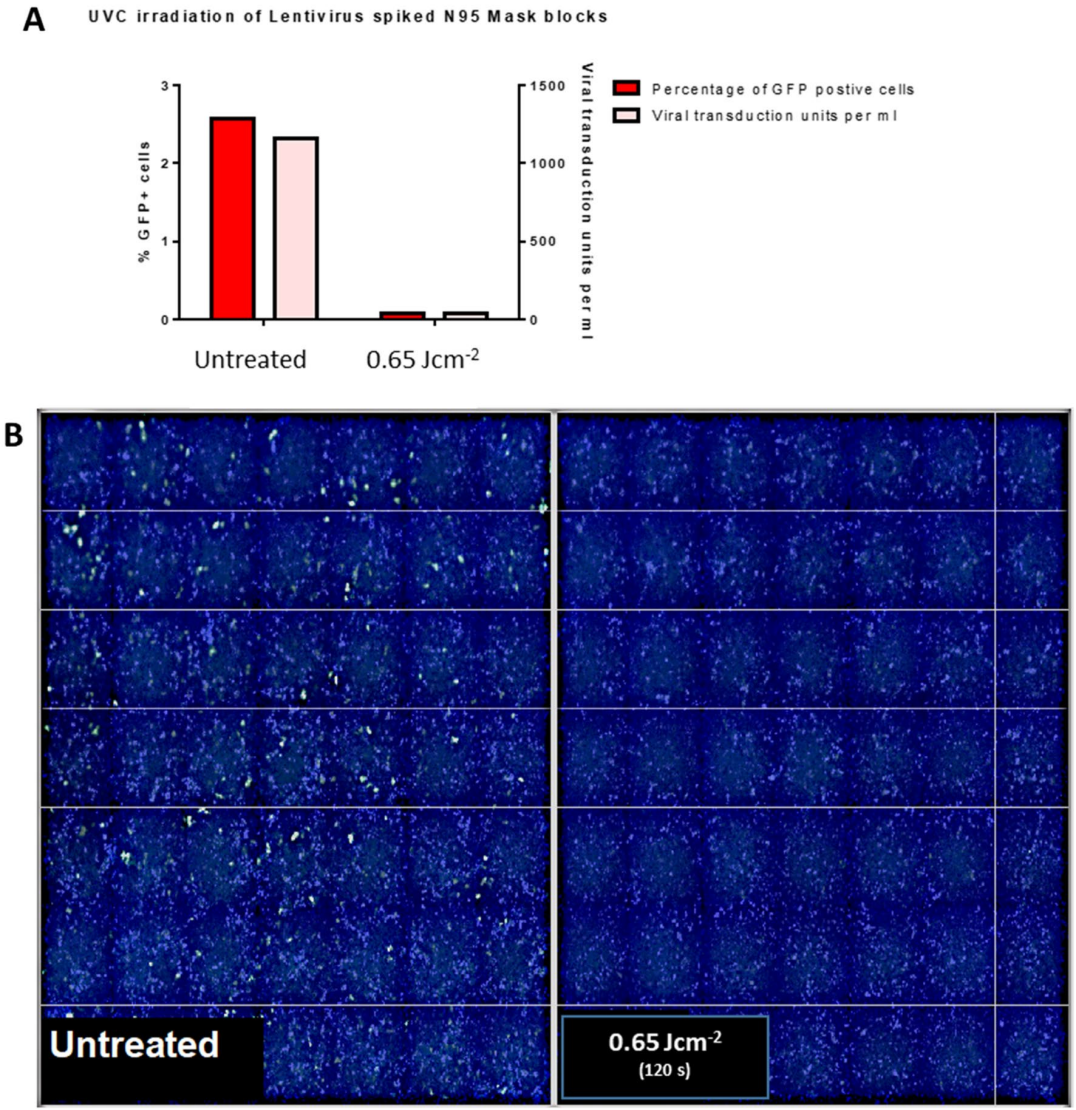
UVC exposure of N95 test coupon squares spiked with LV-EGFP indicated inactivation of lentivirus after treatment for a total of 120 s. N95 test coupon squares (2.5 cm^2^) with all layers were incubated in infection media spiked with LV-EGFP for 15 min. The test coupons were then placed on open 10 cm polystyrene cell culture dishes 15.2 cm from the lamp. After warming up the lamp, the blocks were treated for 60 s per each side. Test coupons were left in infection media for 15 min, protected from light, at room temperature. 0.8 ml of the test coupon derived media mix was used from each condition to infect A549 cells. Cells were fixed with 4% paraformaldehyde, stained with DAPI and imaged using a Cellomics ArrayScan. (**A**) UVC treatment of N95 test coupons spiked with LV-EGFP indicates viral inactivation after 120 s of total exposure. GFP + cells and the total viral transduction units per ml of supernatant were assessed and data shown here are derived from two replicates. (**B**) Fluorescence microscopy analysis of infected A549 cells indicate viral inactivation. Supernatants derived from treated or untreated 3 M-N95 test coupons were used to infect A549 monolayers. 48 h post infection, cells were fixed and stained for nuclei using DAPI (blue).

Supernatants derived from untreated respirator test coupons were used to infect A549 cells and a total of 36001 cells were analyzed. A total of 2.57% cells (approximately 925 cells) were GFP positive because of LV-EGFP infection and constituted around 1155 viral transduction units per ml of analyzed supernatant (Fig. 2A). Supernatants derived from test coupons treated for 60 s each side showed only around 0.08% GFP positive cells (approximately 27 cells out of a total of 33908) accounting for 34 viral transduction units per ml (Fig. 2B). A total of 1.53 Log_10_ reduction was observed.

### Total inactivation of SARS-CoV-2 in spiked N95 apparatuses by 120 s of exposure per side

**Figure 3:**
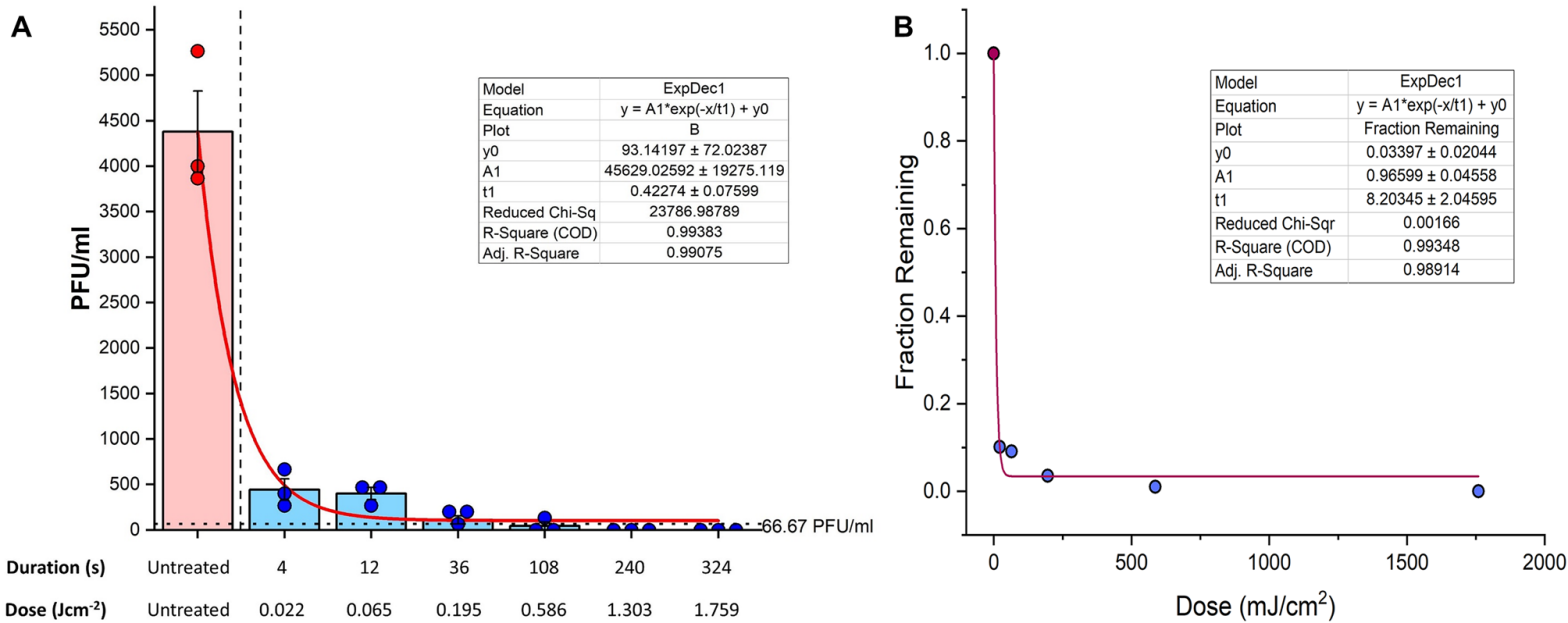
UVC irradiation for 240 s induces over 3.5 log reduction in SARS-CoV-2 viral titers. (**A**) Time course of log reduction of SARS-CoV-2 in N95 test coupons treated with UVC irradiation. N95 mask squares (2.5 cm^2^) with all layers were incubated with infection media spiked with SARS-CoV-2 for 15 min. The respirator coupons were then placed on open 10 cm polystyrene cell culture dishes 15.2 cm from the lamp. After warming up the lamp, the blocks were treated for the indicated amount of time per each side. Respirators were left in infection media at 4 °C overnight. One ml of the mask derived media mix was used from each condition and was titered in VERO-E6 cells using plaque assays targeting the SARS-CoV-2 Spike (S) and Nucleocapsid protein (NP). Each dot represents data from three independent experiments and plotted data bars represent mean and standard deviation for the triplicates. Vertical dashed line delineates untreated and treated parts of the experiment. Horizontal dotted line is the minimal level of detection (approximately 67 PFU ml^−1^). Baseline for the assay is shown using a continuous line. (**B**) Regression analysis of viral reduction as per timely UVC irradiation.

The warmed-up UVC device neutralized approximately 1 log viral titer with as little as 2 s of exposure per side (Fig. 3A). Further reduction of approximately 2 logs was observed by an exposure of 54 s and by 120 s (per side), total inactivation was apparent by the lack of plaque forming units (PFU) derived from the respective experimental settings (T = 120 s per side 3.6 Log_10_ reduction). This was achieved by using an irradiance of at least 5.43 mW cm^−2^. Figure 3 demonstrates exponential decay (r^2^ = 99; p < 0.001) in PFUs and fraction of active virus remaining as function of both exposure times and dose. While we observed a significant reduction by 54 s of exposure per-side, we were unable to replicate it in one of the three independent experiments.

### Total inactivation of non-enveloped EMCV in spiked N95 apparatuses by 120 s of exposure per side

**Figure 4:**
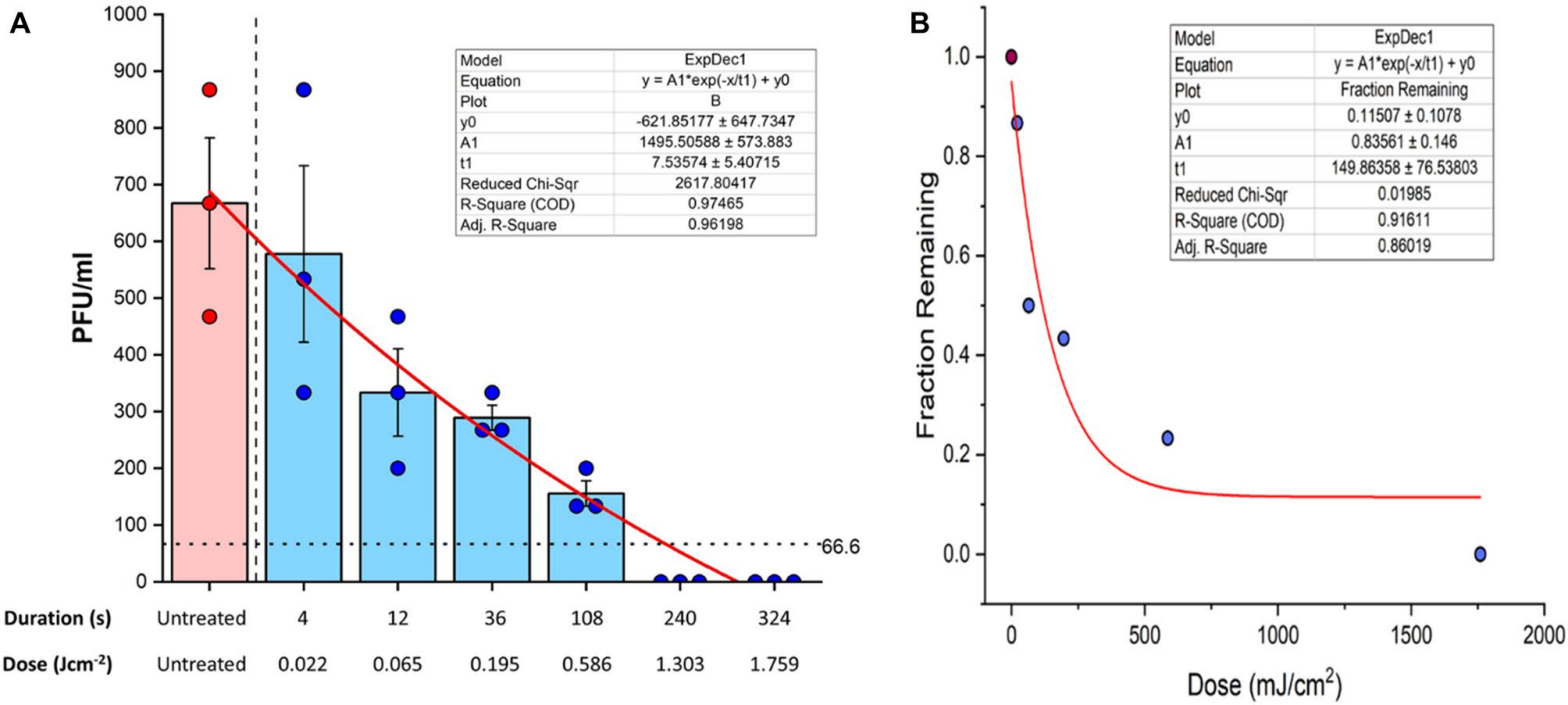
UVC irradiation for 240 s inactivates over 2.5 log reduction in EMCV viral titers. (**A**) Time course log reduction of EMCV PFU in N95 test coupons treated with UVC irradiation. N95 mask squares (2.5 cm^2^) with all layers were incubated with infection media spiked with EMCV for 15 min. The mask coupons were then placed on open 10 cm polystyrene cell culture dishes 15.2 cm from the lamp. After warming up the lamp, the coupons were treated for the indicated amount of time per each side, and were left in infection media at 4 °C overnight. One ml of the respirator derived media mix was used from each condition and was titered in VERO-CCL81 cells using standard plaque assays and crystal-violet staining. Each dot represents data from three independent experiments and plotted data bars represent mean and standard deviation for the triplicates. Vertical dashed line delineates untreated and treated parts of the experiment. Horizontal dotted line indicates the minimal level of detection (approximately 67 PFU ml^−1^). Baseline for the assay is shown using a continuous line. (**B**) Regression analysis of viral reduction as per timely UVC irradiation.

The inactivation kinetics for EMCV was much slower as a function of time (Fig. 4A). A dramatic reduction of PFU for EMCV was seen beyond 54 s of irradiation per side, using an irradiance of at least 5.43 mWcm^-2^ (0.59 J cm^−2^). Nevertheless, exponential decay was observed for EMCV (r^2^ = 91; p = 0.01) in PFUs as well as fraction of active virus remaining as function of dose and exposure time. Complete inactivation (A total of 2.82 Log_10_ reduction) was only observed by 120 s irradiation per side (Fig. 4B).

## Discussion

COVID-19 pandemic caused by SARS-CoV-2 has negatively impacted the entire world, affecting all industries across multiple disciplines. All essential workers, particularly healthcare workers, extensively rely on FFRs such as N95 respirators to minimize the risk of contraction and transmission of SARS-CoV-2. Therefore, such respirators are in high use and demand, with resulting shortages suggesting the need for safe reuse^28^. Based on of SARS-CoV-2 virus survivorship data coupled with the need to preserve FFRs, the USA government recommended the use of one respirator per day, cycling them throughout the week ensuring a minimum of five days between reuse^29^. The risk associated with reuse would suggest further studies to explore the effective decontamination of facepieces. A recent study demonstrated the extensive persistence of SARS-CoV-2 infectious virus in PPE, particularly in FFRs^30^. This has encouraged researchers to evaluate several potential methods for the decontamination of FFRs.

Among the methods of decontamination, ultraviolet germicidal irradiation (UVGI), primarily UVC 254 nm has a lengthy history of antimicrobial applications in healthcare and medical based settings^31^. Due to ease of scalability of UVC based decontamination methods, they are favorable for use during a pandemic. During the current SARS-CoV-2 pandemic UVC air and surface borne decontamination can be deployed more generally, for example to decontaminate public spaces. Here, we evaluated a germicidal UVC 254 nm irradiation system as a potential tool to decontaminate respirators with the aim of assisting their safe reuse.

We first characterized the device by obtaining the dose response parameters as well as identifying the importance of the positioning of the respirators which are to be sterilized to obtain the most efficient decontamination setting (Fig. 1C,D,E). The prototype derivative we had was first used to test the germicidal potential, using a lipid enveloped 3rd generation lentivirus as a model virus under BSL-2 containment. The data suggested robust reduction of lentivirus that were infused within the N95 test coupons upon UVC 254 nm treatment for a total 120 s (Fig. 2; 1.53 Log_10_ reduction). By taking the above data into consideration, we then evaluated a time and dose dependent UVC irradiation protocol for the decontamination of N95 Respirator test coupons spiked with live SARS-CoV-2 under BSL-3 conditions. Of note, two seconds per side of irradiation (4 s total exposure time) indicated the reduction of approximately 1 log of viral titer with a minimal irradiance of 5.43 mW cm^−2^ (0.02 J cm^−2^). While we were able to see an approximate 2.5 log reduction of respirator test coupons treated for a total of 108 s (54 s per side) (0.6 J cm^−2^) in two out of three independent experiments, we were able to see the reduction of approximately 3.6 log viral reduction by 240 s (1.3 J cm^−2^) of total UVC 254 nm irradiation in all experimental replicates. An important note to highlight here is that we used a functional assay such as plaque assay to test the inactivation as opposed to utilizing a technique such as qPCR. Molecular techniques as such may not truly reflect the degree of inactivation given that the presence of nucleic acids such as RNA is not indicative of live virus.

Although we spiked the test coupons with 10^6^ PFU of SARS-CoV-2, we were only able to retrieve an average viral titer of 4.3 × 10^3^ PFU. This can be accounted for in that the spiked test coupons were suspended in a larger volume of 15 ml after spiking. Our study was able to demonstrate a 3.6 viral log reduction by 240 s of irradiation (1.3 J cm^−2^), yet it is currently unknown as to the number of virions that may be present in FFRs derived from real-life settings such as clinics and high biosafety level laboratories. To corroborate our data and to assess the potential of the inactivation device, we used the non-lipid enveloped RNA virus, EMCV in our study. Some investigators have shown non-enveloped viruses although susceptible to UVC damage, may require higher doses or exposure times^32,33^. While the inactivation kinetics were much slower in comparison to the enveloped SARS-CoV-2, total inactivation was observed by 120 s of irradiation per-side (1.3 J cm^−2^), demonstrating the ability of the device to decontaminate both enveloped and non-enveloped viruses. Previous studies have looked at UVC 254 nm treatment in viral species and reported data suggesting lentiviruses (*Retroviridae*) although lipid-enveloped, show unusually high level resistance for UV-C inactivation (D_37_ = 67–110 J m^−2^; D_90_ = 280 J m^−2^)^23,34,35^. Conversely, D_90_ and D_37_ values for SARS-CoV-2 (*Coronaviridae*) has been determined to be 23 J m^−2^ and 3 J m^−2^ indicating the increased susceptibility to UVC 254 nm in comparison to other viruses we have used and is consistent with previous studies^23,30,32,34,35^. Additionally, the D_90_ and D_37_ values for EMCV (*Picornaviridae*) has been determined as 55 J m^−2^ and 44–53 J m^−2^, suggesting a slight increase in resistance to UVC 254 nm in comparison to SARS-CoV-2 but not as much as the lentiviruses^23,34,35^. In summary, our data is consistent with previously reported data indicating susceptibilities for different viral species to UVC 254 nm inactivation and its potential application for PPE decontamination.

While there are promising data in the current study, several limitations warrant further investigation. In this study, instead of full N95 respirator, we directly exposed each side of the respirator test coupon to UVC 254 nm at a series of doses related to the respirator re-use device conveyor speed. This was for practical reasons, as the full respirator irradiation system could not be moved into the BSL-3 lab. Since all UV exposure is direct line-of-sight we anticipate the curvature would not impact the result in the full respirator irradiation system, as the respirators are irradiated simultaneously from both sides and from various angles as the masks move on the conveyor system in between the UVC 254 nm irradiation sources. Further the respirator material is not reflective, and a significant amount of the radiation will scatter into the respirator filter material; therefore, for these reasons we expect the test coupon irradiation to be representative of the actual respirator exposure on the conveyor system.

N95 manufacturers such as 3 M do not recommend the decontamination as well as the reuse of FFRs. This probably has been suggested based on a study conducted by 3 M in which respirators were exposed to cumulative dose of 10 J cm^−2^ between five to 10 cycles resulting in changes in Respirator fit and physical characteristics^36^. This is indicative of the fact that FFRs are only able to withstand a certain amount of decontamination cycles before the performance is compromised. Some studies have suggested that repetitive donning and removal FFRs result in impaired respirator fit^37^, indicating that such respirators can only be reused a very small amount of time regardless of the decontamination efficiency. While there are no strict guidelines explaining the possible number of reuses for FFRs, the CDC recommends no more than 5 reuses based on two studies^37–39^. Therefore, a non-deleterious germicidal protocol can be implemented for the decontamination of FFRs to support the safe reuse for a limited number of times. Other studies have demonstrated the effects on the materials and fit upon UVC irradiation using doses 1–1.2 J m^−2^. It was noted that there was no significant damage to the respirators, compromising the performance. However, some impact on fit due to strap degradation was noted after an unspecified number of cycles and was not quantitatively addressed^16,19,22,26^. Furthermore, a variety of N95 FFRs in the range considered here (1–1.2 J cm^−2^) were tested for performance post treatment and found no significant respiratory filter material damage, impact on filtration performance. The proposed device in the current study will be operating below these doses as lower end and near these dosages at the upper exposure ranges.

The current COVID-19 pandemic has presented a multitude of challenges in healthcare settings and the seemingly apparent shortage of the PPE such as FFRs is currently an issue as such. This study evaluated UVC based germicidal decontamination protocol which has the potential to inactivate 3.6 log SARS-CoV-2 within a total time frame of 240 s, facilitating a potential avenue for the safe reuse of the FFRs.
